# Use of NeuroEyeCoach™ to Improve Eye Movement Efficacy in Patients with Homonymous Visual Field Loss

**DOI:** 10.1155/2016/5186461

**Published:** 2016-09-15

**Authors:** Arash Sahraie, Nicola Smania, Josef Zihl

**Affiliations:** ^1^School of Psychology, University of Aberdeen, Aberdeen, AB24 3FX, UK; ^2^Neuromotor and Cognitive Rehabilitation Research Centre, Department of Neurological and Movement Sciences, University of Verona, Verona, Italy; ^3^Neurorehabilitation Unit, Borgo Roma University Hospital, P.le L.A. Scuro 10, 37134 Verona, Italy; ^4^Department Psychology, LMU University of Munich, Leopoldstrasse 13, 80804 München, Germany

## Abstract

Visual field deficits are common in patients with damaged retinogeniculostriate pathways. The patient's eye movements are often affected leading to inefficient visual search. Systematic eye movement training also called compensatory therapy is needed to allow patients to develop effective coping strategies. There is a lack of evidence-based, clinical gold-standard registered medical device accessible to patients at home or in clinical settings and NeuroEyeCoach (NEC) is developed to address this need. In three experiments, we report on performance of patients on NEC compared to the data obtained previously on the earlier versions of the search task (*n* = 32); we assessed whether the self-administered computerised tasks can be used to monitor the progress (*n* = 24) and compared the findings in a subgroup of patients to a healthy control group. Performance on cancellation tasks, simple visual search, and self-reported responses on activities of daily living was compared, before and after training. Patients performed similarly well on NEC as on previous versions of the therapy; the inbuilt functionality for pre- and postevaluation functions was sensitive to allowing assessment of improvements; and improvements in patients were significantly greater than those in a group of healthy adults. In conclusion, NeuroEyeCoach can be used as an effective rehabilitation tool to develop compensatory strategies in patients with visual field deficits after brain injury.

## 1. Introduction

We explore our surrounding environment by moving our eyes on average three times per second. The eye movement episodes are punctuated by brief periods (100–300 ms) of fixations. This pattern of activity ensures detailed image processing by the high density cone-receptor region of our central vision [1]. The resultant continuous perception of the stable world relies on amalgamation of lower resolution peripheral vision with high resolution central information in a spatiotopic frame of reference [2]. This dynamic process encompasses the suppression of noise or distractors and selective enhancement of target objects [3]. The selection of candidate targets for subsequent eye movements (saccades) is achieved through a combination of stimulus driven bottom-up and goal driven top-down mechanisms [4].

Visual field deficits often accompany lesions of the visual pathways which in turn disrupt the selection of targets falling within the impaired visual fields [5]. Abnormal patterns of eye movement are reported in approximately 60% of such cases [6]. One method for quantifying disturbances of visual processing is to make use of a visual search paradigm where the patient is required to report the presence or absence of a target amongst distractor items, often but not exclusively, presented on a computer screen [7]. The reaction times are then compared to those for target detection in the sighted field in the same individual or in a group of healthy individuals. The inverse of the slope for a linearly fitted plot of reaction times as a function of the number of distractor items reflects “search efficiency” [8]. In general, for healthy adults when targets and distractors are easily discriminable (pop-out search), the slope is shallow (high efficiency), but steeper slopes are expected when targets and distractors share features (complex or conjunction search).

Eye movement recordings of patients with visual field deficits following brain injury reveal a number of characteristics [9]. These include smaller saccade amplitudes, and, hence, a larger number of fixations; limited exploration of the contralesioned visual field; and more between-hemifield saccades often summarised as disorganised eye movements leading to slower reaction times for targets in contralesioned hemifields. Disturbances of eye movement dynamics are also reported in the sighted (ipsilesioned) hemifield [6, 10].

In clinical practice, the rehabilitation of patients with visual field deficits is often conducted by occupational therapists or low-vision experts. The aim of any intervention is to improve the patient's interactions with their immediate surrounding and increasing their confidence in tasks such as shopping or commuting. The use of computerised visual search tasks as a rehabilitation tool to improve eye movements after brain injury was first reported in a group of 30 patients [11]. Patients were given systematic practice with large saccadic eye movements to search for targets presented at unpredictable positions in both the affected hemifield and the entire field of gaze. This class of treatment was later extended by use of a visual search paradigm to improve scanning strategy. Simultaneous recording of eye movements in a group of 60 patients provided further evidence for spatially disorganised pattern of eye movements in 60% of cases [6], with improved visual scanning in all 13 cases that underwent visual search training. With better use of the remaining sight as well as efficient search strategy, patients were able to compensate for their partial blindness; hence, the technique has been termed* compensatory*. This technique with various modifications has been used in 14 studies to date, with a total of 593 patients with homonymous visual field loss and persistent visual disabilities (see Table 1). Indeed a recent systematic review [12] has identified eye movement training as the most promising approach to visual rehabilitation in stroke patients.

The main outcome has been a significant improvement in visual search performance accompanied by more efficient oculomotor strategies and a reduction in visual disability as assessed with standardised questionnaires and behavioural measures. The treatment effects remained stable and persisted after 4–12 weeks of follow-up [18, 19]. The efficacy of this treatment approach in improving visual search has been compared to a number of other methods, and although the findings may not be definitive, they appear to show that the visual search training is better than reading specific training [21], nonspecific visual training [17], standard occupational therapy [20], or counselling with regard to coping strategies [19]. Importantly, time since brain injury [19] and age of hemianopic patients [22] did not play a significant role in the treatment effect. More recently, a compensatory technique based on tracking a moving target at a constant speed with a sudden change in its position has also shown improved eye movement behaviour and faster detection for targets presented in the blind field [25]. A follow-up study of 78 hemianopic patients showed significant improvements in eye movements as well as in activities of daily living after 11 days of training [26]. In one study [23], the level of improvements in an eye movement training task appeared to be similar to that of an attention task used as a control. However, in a later study of 52 patients [24], the revised version of the task combining search and reading showed significantly more improvements in search time and reading speed versus a control task.

We have developed NeuroEyeCoach (NEC) with the aim of providing a standardised protocol for clinical management of patients using a compensatory technique. The program systematically increases the task difficulty from a pop-out to more complex search and finally conjunction searches by manipulating distractor number and target/distractor similarity. We have ensured that this instrument is appropriate for use under supervision in clinical settings as well as being accessible for home use. To facilitate wider access to therapy, it is Internet deliverable and is self-adaptive by systematic adjustment of the time allowed for the visual search to reach predefined level of accuracy.

Here, we report on three observational studies. In the first study, we report on the comparison of outcomes in patients using the NEC (*n* = 16) compared to the data obtained previously using the earlier versions of the therapy [19], showing that NEC leads to a comparable level of improvement. In the second study, we show evidence that the patients also show improvements on assessments included in NEC (*n* = 24) and, in a final study, we compare the changes in visual search in a group of patients (*n* = 9) with those of a similar age control group (*n* = 10), when both groups attended clinical rehabilitation units and conducted the therapy under supervision. The findings show that the level of improvement is greater than the effect of practice in healthy controls.

## 2. Experiment  1

In this observational study, we compared the performance on two outcome measures of 16 patients who had undergone compensatory therapy using NEC, supervised and in clinical settings (experimental group), and 16 matched patients who had delayed treatment (waiting group) and served as a control group but were assessed three times as the experimental group. Patients using NEC were assessed for the fourth time; however, the control group commenced the therapy shortly after completing assessment 3. For the delayed treatment group, we used previous data from our database of 297 patients and selected patients who matched closely to the treatment group. The groups were matched with respect to age, time since onset of hemianopia, side and extent of hemianopia, visual field sparing, and degree of impairment with respect to search performance. The two outcome measures were time taken to complete a pen and paper version of the cancellation task and reaction times on a visual search task ([19, pages 78 and 81]). Both outcome measures were repeated at 4 time periods outlined below. The findings were also compared to previously reported data [19], in a larger group of patients using an earlier version of the visual scanning training program.

### 2.1. Participants

Thirty-two patients (4F; mean age: 56.6 years; range: 17–82 years) with either left- (*n* = 16) or right-sided (*n* = 16) homonymous hemianopia were monitored. No patients exhibited signs of visual neglect or suffered from depressive symptoms. Those with moderate to severe difficulties with attention or memory were excluded. Aetiology of brain injury causing hemianopia was a left- or right-sided posterior artery infarction. Mean visual field sparing was 2.5 degrees (SD = 1.3 degrees; range: 1–6 degrees). Time between onset of hemianopia and first assessment ranged from 10 to 64 weeks, with an average of 25.1 weeks (SD = 11.2, range: 10–48 weeks). The first assessment was followed by a training-free interval (“waiting period”), with an average duration of 8 weeks (range: 6–9 weeks). Training took approximately 2 weeks and a follow-up assessment (assessment 4) was performed 11 weeks on average (range: 8–16 weeks) after the completion of therapy.

### 2.2. Methods

#### 2.2.1. Assessment Measures

Apart from quantitative visual field mapping with a Tübingen perimeter, patients underwent a comprehensive ophthalmologic and neuropsychological examination to ensure that they exhibited no additional visual, oculomotor, or cognitive problems. Visual near and far acuity was at least 0.8 on Snellen scale (0.1 logMAR).

For assessing visual exploration performance before and after treatment, we used the same standardised cancellation task in parallel versions, which we have found sensitive to changes in visual exploration performance in earlier studies (Schuett et al., 2012 [21] and Zihl, 2011 [19]). The test consisted of 20 black diamonds (targets) randomly embedded in 22 black dots and crosses (distractors) on a sheet of white paper. At a viewing distance of 30 cm, the stimulus array is subtended 44.6 deg horizontally and 35 deg vertically; stimulus size (diameter) was 0.8 deg. Patients were not informed about the number of targets but were asked to mark all targets with a pen as accurately and as quickly as possible with their right hand. Visual exploration performance was defined as the time required performing the task, as well as the number of errors. In addition, we used a standardised questionnaire to assess subjective experiences before and after treatment similar to those reported earlier [19]. The questionnaire included three items (vision too slow, bumping against obstacles, and getting lost) and three response categories for each item (no/mild difficulties, moderate difficulties, and severe difficulties). All the experiments reported were conducted in accordance with the Code of Ethics of the World Medical Association (Declaration of Helsinki) and informed consent had been obtained from all participants for the use of their data for research purposes.

#### 2.2.2. Intervention Method

Intervention was performed with NEC in clinical settings, according to a set procedure. During the initial software installation, the program uses screen resolution data and the length of a 400-pixel horizontal and vertical line, to calculate the screen dimensions and viewing distance needed such that the screen subtends a minimum of ±20° in horizontal extent. Chin-head-rests were not used in any of the studies reported; however, for measurements conducted in the clinic, the supervising therapist controlled the viewing distance. Those patients attending clinics also followed the on-screen instructions and the supervising therapist did not interfere in anyway with the patient's progress. As NEC makes use of alphanumeric characters, all users are initially screened to ensure that they can comfortably see the items on the screen, starting from a character size subtending 1.4°. The size was increased in 0.1° steps until the letters were clearly seen up to an upper limit of 1.8°. The therapy program contains 12 levels, with 4 levels at each of pop-out, complex, and conjunction search categories. At each level, the task difficulty was altered by setting the set-size to either 8, 16, or 24 resulting in 3 discrete sublevels. Examples of pop-out search include searching for either a T or an X amongst Os or an H amongst Cs. Complex searches include searching for an S amongst Cs; an O amongst Gs; or a B amongst Ds. Both target shape and colour were altered in conjunction searches (searching for green X, amongst blue Xs and green Rs; a green b amongst blue bs and green ps; or a green T amongst blue Ts and green upside-down Ts). Target and distractors were equally distributed on the left and right half and the upper and lower parts of the screen and an example of the target item was always presented in the middle of the screen within an orange coloured circle. Progression to the subsequent level was contingent upon achieving 85% or higher in accuracy in at least 2 of the 3 sublevels. Each sublevel took approximately 15 minutes to complete and consisted of 200 trials with targets being present in 100 trials. The patient's task was to indicate whether or not a specific target was present by pressing one of two mouse buttons. The time allowed for each trial was limited (1500 ms) but increased by 500 ms, if a level had to be repeated (i.e., performance below 85% correct in 2 sublevels). Unlimited time for response was provided, if the patient failed to achieve the accuracy threshold for passing a level for the second time. Patients participated NEC-training on a regular basis, with at least 5 sessions per week. Sessions lasted on average 45 min (sufficient to complete one level), with short (1-2 min) breaks after approximately 15 min, depending on their level of fatigue/alertness.

### 2.3. Results

All data were analyzed using IBM® SPSS® Statistics 20. Demographical and clinical data were statistically analyzed using *χ* ^2^-test for gender and side of hemianopia and *t*-tests for independent groups with a significance level of *p* ≤ 0.05. Statistical data analysis of time taken to complete the cancellation task and visual search reaction time for target present trials before and after treatment (experimental group) or waiting period (control group) of the total group of patients was performed using a two-factorial ANOVA with repeated measurements, Greenhouse-Geisser correction, and a significance level of *p* ≤ 0.05. Statistical comparisons of the time taken to complete the cancellation task and visual search reaction time for target present trials within and between groups were performed with *t*-tests and all reported *p* values of these two analyses are Bonferroni corrected at *p* ≤ 0.02.

Subjects in the experimental and control groups did not differ significantly with respect to sex (*χ* ^2^(1) = 0.00, *p* = 1.00), age (*t*(30) = −0.31, *p* = 0.759), side of hemianopia (*χ* ^2^(1) = 0.00, *p* = 1.00), visual field sparing (*t*(24.73) = 1.57, *p* = 0.130), and time since injury (*t*(30) = −0.55, *p* = 0.586). Mean number of training sessions to complete NEC was 13 (range: 9–20).

Regarding the cancellation task, ANOVA showed a significant main effect for repeated measurements (*F*(1,30) = 56.83; *p* < 0.001) but not for group (*F*(1,30) = 3.51; *p* = 0.071). The interaction between repeated measurements × group was significant (*F*(1,30) = 48.54; *p* < 0.001). ANOVA for visual search revealed similar effects: there was a significant main effect for repeated measurements (*F*(1,30) = 75.67; *p* < 0.001) but not for group (*F*(1,30) = 1.57; *p* = 0.221). The interaction between repeated measurements × group was significant (*F*(1,30) = 57.06, *p* < 0.001).

Table 2 shows the results of performance of the various assessments for the experimental and the control groups. In the experimental group, there was a small (−3.0 s; 6%), significant (*t*(15) = 4.01; *p* < 0.001) change in time taken to complete the cancellation task between the first and second assessment (waiting period); no significant change was found in the visual search task (*t*(15) = 1.03; *p* = 0.320). After training (assessment 3), we found a significant (*t*(15) = 7.93; *p* < 0.01) decrease in search time in the cancellation task (~31%) and in the visual search task (~27%; *t*(15) = 8.90; *p* < 0.01). Accuracy was perfect (100%) in 9 patients at assessment 2 (before treatment); the rest (*n* = 7) omitted 2 targets on average in either task (range: 1–6). In assessment 3 (after training), 12 patients showed perfect accuracy in both tasks, while four patients omitted still some targets (M = 1.5; range: 1–3). Performance in both tasks did not differ significantly between assessments 3 and 4 (after treatment and at follow-up) (cancellation: *t*(15) = −1.15; *p* = 0.267; visual search: *t*(15) = 1.27; *p* = 0.224). In the control group, we did not find significant changes in reaction times at assessments 1 and 2 (before and after the waiting period) in the cancellation task (*t*(15) = 0.73; *p* = 0.478) and visual search task times (*t*(15) = 1.4, *p* = 0.181). It is important to note that experimental and control groups did not differ significantly at assessments 1 and 2 (before treatment/waiting period), either on time taken to complete the cancellation task (*t*(30) = −1.17, *p* = 0.251) or for visual search times (*t*(30) = −0.40, *p* = 0.694).

At assessment 1 (before the waiting period), cancellation and visual search performance was accurate (100%) in 10 patients; the rest omitted 1 target on average (range: 1–3). At assessment 2 (end of the waiting period), 9 patients performed accurately, while the rest (7 patients) omitted 1 target on average (range: 1–4).

Subjective reports support the observed positive outcome in scanning (see Table 3). At assessment 2 (before treatment), all hemianopic subjects (100%) reported at least moderate difficulties; at assessment 3 (after treatment), the rate decreased to 40% (4 subjects); at assessment 4 (follow-up), the rate was 12.5% (two subjects). The most frequent response was “vision is too slow,” followed by “bumping into objects” and “getting lost.” In contrast, the patients in the control group at assessment 3 compared to assessment 2 reported a much lower decrease in vision related difficulties (100% versus 81.3%).

For a better “grading” of the results of this study, we have placed the findings qualitatively in the context of the outcome measure previously obtained using a similar method of systematic eye movement training in a larger group (*n* = 117) of hemianopic subjects [19]. The outcome is similar with respect to search times in both tasks. In the earlier study, improvement in scanning speed in the cancellation task was ~43% and in the visual search task ~25%, respectively compared to 31% and 27% reported here (accuracy was also comparable). The smaller number of training sessions in the earlier study (10 versus 13 on average) can be explained by the fact that, in the earlier version of scanning training, the therapist decided when the next level was reached on a more variable criterion.

In summary, the findings show that NEC can lead to improved visual search in patients with partial blindness after brain injury. In addition, the improvement in visual scanning as assessed with cancellation and visual search tasks strictly depended on systematic treatment, because no significant changes in performance in the two tasks were found in the waiting group. In addition, the overall effect of using NEC appears to be similar to the previous compensatory therapy based on visual search with the added advantage of having set criteria for progression to different levels of difficulty.

## 3. Experiment  2

We have established that, similar to those patients using the previous versions of the program, those undergoing eye movement training by use of NEC also show improvements assessed using the previously reported outcome measures (visual search times and pen and paper based cancellation task). Here, we report on an additional group of patients (*n* = 24) undergoing eye movement training using NEC. This time the outcome measures used to assess the therapy related changes were an integral part of the NEC. Therefore, patients completed the pre- and post-assessments included in the NEC program on a computer and unsupervised. These included a computer-based version of the cancellation task and a standard visual search task as well as filling out an activity of daily living questionnaire (ADL, described below). This ADL has been developed specifically for this patient group and the findings previously reported in the literature [14, 27]. In this observational study, we aimed to determine if this additional cohort of patients also benefits from visual search training and whether the changes are measurable using the incorporated pre- and post-assessments.

### 3.1. Participants

Twenty-four participants (8F; mean age 58.5; SD = 17.9; range: 15–80) made use of the NeuroEyeCoach program either at home (15 cases) or within a rehabilitation clinic (9 cases). The participants had a visual field defect on the left (*n* = 8), right (*n* = 12), or both hemifields (*n* = 4; i.e., bilateral upper or lower quadrantanopia). The time between the onset of the field defect and the first training session ranged from 3 to 170 months with an average of 45.5 months (SD = 48.6).

### 3.2. Methods

*Pre- and Post-assessments*. NeuroEyeCoach program incorporates a series of baseline assessments, in order to quantify the scanning behaviour. The visual search performance was measured for detection of a black O amongst black Ts and Ls. For 10 trials, patients were asked to report the presence or absence of a black O amongst 4 distractors (2Ls and 2Ts) using either of two mouse buttons (or left and right arrow keys). They then completed 4 blocks of 20 trials at set-sizes of 4, 8, 16, and 24. The pre- and post-training RT figure are the mean of median reaction times from all blocks. The total number of errors in all blocks is also used to compare pre- and post-training errors. During the cancellation task, patients were shown three different screens each containing 20 targets (e.g., diamonds) and 23 non-targets (e.g., 13 circles and 10 stars). The task was to click the mouse button on targets and press the space-bar when they had found all targets. When all the targets were highlighted, the timer would stop and the next set of instructions would appear. The cancellation task score is the median of the three measurements. This task was also repeated after training. Patients also reported their perceived disability on a 5-point scale for performing various activities of daily living. The nine questions were difficulties seeing obstacles; bumping into obstacles; losing their way; finding objects on a table; finding objects in a room; finding objects in a supermarket; crossing the road; using public transport; or using a computer. The rating scale ranged from no difficulty at all (1) to occasional (2), sometime (3), often (4), or having severe difficulties (5). Patients performed all the assessment tasks once again after completing the NeuroEyeCoach program.

### 3.3. Results

The study is of repeated-measure design with 5 factors of scores on cancellation task, errors on visual search, visual search reaction time, reaction time for target absent trials, and self-reported disability, all being obtained twice, once before the start of the training and once after completing training.

The duration of training for those completing the program in the clinical setting or online was on average 13.1 (SD = 1.76) and 14.0 (SD = 1.96) sessions, respectively.

Patients rated their perceived problems with performing activities of daily living significantly less after the training (M = 13.21, SD = 5.34) than before the training (M = 18.47, SD = 6.63) (*t*(18) = 4.381, *p* < 0.001, both latter measures significantly higher than the critical *t*-value for Bonferroni correction (*t* = 2.574)).

Paired-sample *t*-tests were conducted to compare the performance before and after the therapy on the time taken to complete the cancellation task, number of errors in visual search, and the disability score. The time taken to perform the cancellation task on the computer screen before (M = 44.5 s, SD = 38.5) and after (M = 41.6 s, SD = 35.3) training was not significantly different (*p* = 0.267). However, patients made significantly fewer errors after the training (M = 3.42, SD = 4.47) than before training (M = 9.17, SD = 5.76), *t*(23) = 4.848, *p* < 0.001 on the visual search task.

**Figure 1:**
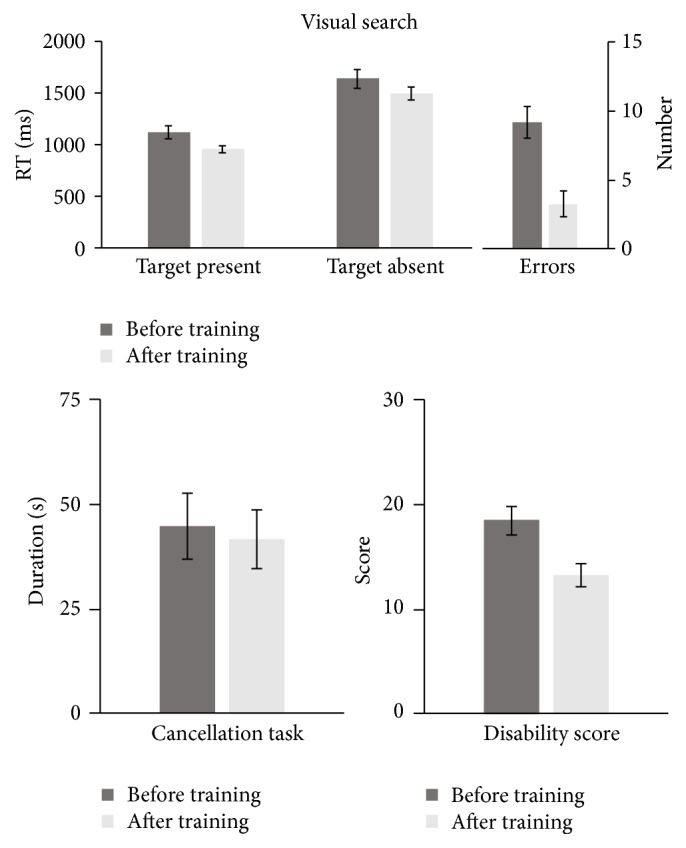
A summary of patients' data on tasks conducted before and after training. All dark and light bars represent pre- and post-training data, respectively. Error bars plot ±SEM.

Performance on the reaction times (RT) on visual search tasks was analyzed using a 2 × 2 repeated-measure ANOVA with a within subject factor mean RT (2 levels, in target present trials and in target absent trials) and Time (2 levels, before and after training). There were significant main effects of RT (*F*(1,23) = 167.6, *p* < 0.001) and time (*F*(1,23) = 11.48, *p* = 0.003) but no significant interaction (*F*(1,23) = 0.312, *p* = 0.581). Paired-sample *t*-tests indicated that post-training visual search times (M = 957 ms, SD = 182) were faster than the pre-training (M = 1125 ms, SD = 304), *t*(23) = 4.167, *p* < 0.001. Patients' reaction times in “no target present” trials was also significantly shorter after the training (M = 1491 ms, SD = 312) than before (M = 1637 ms, SD = 428) (*t*(23) = 1.795, *p* = 0.022). Both reaction time parameters show that patients are significantly faster after training on the visual search task. It is also noteworthy that they also made significantly less errors in the visual search task; therefore, the improvements in reaction time cannot be attributed to the speed-accuracy trade-off. A summary of the findings discussed above is shown in Figure 1. A better visual search strategy may underline the improved speed in detecting a target in target present trials during visual search. However, the reasons for improvements in reaction times to report the absence of a target are more complex. Of course, it is possible that, following systematic training, patients are faster in searching the entire screen for a target and therefore also improve in reporting its absence in the target absent trials. However, as the reaction time in target absent trials can also be affected by other factors such as subjective bias and confidence, it may also be the case that, following practice and over repeated exposures, the patients are more confident (hence faster) in reporting the absence of a target.

In summary, the findings show that this cohort of patients also benefitted from eye movement training. The training led to faster visual search times, reduction in the number of errors made, and improvements in reported activities of daily living. The computer-based cancellation task, however, was not sufficiently sensitive to detecting the behavioural changes.

## 4. Experiment  3

We have demonstrated that, similar to the previously reported findings, systematic training on visual search tasks can lead to shorter search times and improved responses on activities of daily living in patients with partial blindness subsequent to brain injury. In almost any psychophysical task, the performance can improve with repeated practice. The extent of such perceptual learning in normal observers is often limited. This is due to the fact that one may consider that the normal observers already perform at optimum. As the patients also have a much slower search time after brain injury, the baseline measures between the patients and normal observers differ significantly. One way of comparing the effect of training in the two groups would be to normalise the improvements as a fraction of the baseline performance. In a third experiment, we have compared the level of improvements in search times of a group of patients with those of healthy controls of a similar age to investigate the extent of changes in both cohorts.

### 4.1. Participants

In order to ensure that both patients and normal controls conduct the training under similar conditions, we have compared performance of all those patients who attended a rehabilitation clinic on daily basis (*N* = 9) and their data was included in Experiment 2 with those of a similar age range healthy controls who also attended a rehabilitation clinic again on daily basis. We recruited 10 healthy observers (5F, mean age 60.5 years, SD = 9.5, and range 47–77) and their data compared to the group of 9 patients (2F, mean age 58 years, SD = 19.1, and range 26–80). For recruitment of the control group, ethical approval was granted by the ethics review, School of Psychology, University of Aberdeen, and informed consent was obtained from all participants.

### 4.2. Method

Healthy observers attended the Neuropsychological Day Clinic at the Max Planck Institute of Psychiatry in Munich (Germany; same as for Experiment 1) for five days per week and were paid for their participation. They received the same instructions as those of the patient group. The healthy control group and patients underwent the same training by completing NEC on the same apparatus and clinical settings. All participants completed the pre- and post-assessments on the online version of NEC (as described in Experiment 2) and the control group did not report any disability.

### 4.3. Results

Performance on the reaction times (RT) on visual search tasks was analyzed using a 2 × 2 repeated-measure ANOVA with a within subject factor, mean RT (2 levels, in target present trials and in target absent trials) and time (2 levels, before and after training). There were significant main effects of RT (*F*(1,8) = 150.6, *p* < 0.001) and time (*F*(1,8) = 14.12, *p* = 0.006) but no significant interaction (*F*(1,8) = 2.50, *p* = 0.153). After completing NEC, patients had significantly faster search reaction time (M = 1041 ms, SD = 134) than before training (M = 1357 ms, SD = 273) (*t*(8) = 5.09, *p* = 0.001). Reaction times for reporting target absent trials after the training (M = 1645, SD = 176) were also shorter than before training (M = 1894, SD = 340) (*t*(8) = 2.73, *p* = 0.026), but this is below critical *t*-value for Bonferroni multiple comparison (critical *t*(8) = 3.206; 4 comparisons).

Similarly, healthy controls also had faster visual search times after training (M = 598 ms, SD = 103) than before (M = 666, SD = 106) and this difference was significant (*t*(9) = 3.53, *p* = 0.006). However, their reaction times in target absent trials (M = 754, SD = 147) were not significantly shorter after than before the training (M = 842, SD = 202), (*t*(9) = 1.32, *p* = 0.22).

As both patients and controls improved in search times following training, we were interested to establish if the magnitude of improvements was comparable between the two groups. Overall, the patient group improved more (M = 313 ms, SD = 184) than the control group (M = 68 ms, SD = 60). An independent-sample *t-*test was conducted to investigate whether this difference was significant. Levene's test for equality of variance showed that the two variances were significantly different (*F* = 8.643, *p* = 0.009); therefore, the degrees of freedom for the independent-sample *t*-test were adjusted accordingly. The patient group showed significantly more reduction of their search times than the control group (*t*(9.56) = 3.802, *p* = 0.004). It is, however, important to note that the visual search times were significantly longer (both before and after training) in the patient group compared to the normal controls. Therefore, we have accounted for this disparity by calculating the improvement in reaction times as a percentage of the visual search time before training. This analysis also showed that the patient group improved significantly more (M = 21.8%, SD = 9.8%) than the control group (M = 10.1%, SD = 8.3) (*t*(17) = 2.82, *p* = 0.012).

The following observations may also be noteworthy. For the group of 9 patients, we also analyzed the visual search times in trials where the target was present in their sighted field compared to the blind field. The search times for sighted field presentations in the patient group before the training (M = 1182, SD = 216) and after training (M = 944, SD = 130) were much slower than those for normal controls, further emphasising that the processing in the normal hemifield is also affected after brain injury. However, on average, patients made less improvements in their sighted field (M = 17.7%, SE = 6%) compared to their blind field (M = 25.1%, SE = 4%), although this difference is not significant, probably due to small sample size.

## 5. Discussion

We have reported on the outcome of NeuroEyeCoach (NEC), designed to be used both in clinical settings and by patients in the home environment. In a series of three experiments, we have shown that improvements in visual search following NEC are similar to those reported in earlier versions of the program [19]. The findings from Experiment 2 indicate that NEC is an effective compensatory approach for those with homonymous visual field loss and improved subjective activities of daily living. The absence of a significant improvement in the NEC cancellation task may be related to the degree of difficulty that patient may experience in using a computer mouse to click on objects on a screen compared with conventional pen and paper version. Therefore, in clinical practice, the pen and paper task may be a more appropriate measure to monitor the improvement. Also, it is likely that this shortcoming might be bypassed by using a touch sensitive screen for recording pointing responses to visual items and we intend to test this in further studies. Finally, Experiment 3 showed that the use of NEC in patients can lead to more than twice the magnitude of improvements compared to normal controls, even when accounting for the overall noisier and slower performance of the patients. It should be noted, however, that improvements in patients and normal subjects might be qualitatively different. While normal subjects might benefit from an improved preexisting scanning strategy in terms of speed, patients with homonymous visual field loss benefitted because they regained an effective scanning strategy to substitute for the lost visual field, which is a crucial prerequisite for grasping the actual surrounding with high accuracy and speed. It is important to note that the visual search times in patients still remained slower than the healthy controls. Further studies are required to investigate both the limits of recovery and the factors affecting its extent.

Compensatory approaches to visual rehabilitation in partially sighted patients are aimed at increasing eye movement efficiency, allowing the patients to better explore their environment and to make the most of their remaining sighted field. A large body of evidence (see Table 1), including randomized control trials [17, 20, 24] has shown that patients benefit from systematic eye movement training. In Lane et al. [23], patients' improvements were not significantly more than those found after an attention training task; however, results in line with other investigations were found in their later study [24]. Almost all previous studies of compensatory therapies make use of visual search tasks. Use of visual search in improving search efficiency of hemianopic patients was first reported in 1988 [11]. Other than its use in various forms in a limited number of rehabilitation clinics over the past 25 years, there has been a marked lack of availability of an effective evidence-based gold-standard registered medical device accessible to patients at home or in clinical settings. In clinical practice, this void has been filled by using a number of devices that originally had been designed to address other problems, hence being suboptimum for rehabilitation of vision loss. Devices such as Dynavision (dynavisioninternational.com) or Sanet Vision Integrator (SVI, svivision.com) were originally designed for improving athletes' performance on visuomotor tasks, effectively enhancing their reaching behaviour. For example, in Dynavision, training consists of asking an individual to reach out to a lit-target on a large board, a task similar to a simple pop-out search. In the case of SVI, a more elaborate set of targets can be reached as the device makes use of computer display technology; nevertheless, in both cases there is an absence of a set of systematically developed protocols, specific to visually impaired patients. An effective intervention would need to be adaptable to the patient's performance such that the task difficulty is altered to encourage the patient to improve on the task. Also, should the patient find the task excessively difficult, it would need to be systematically modified to avoid fatigue and despondency. We have addressed both these points in the design of NEC. The criterion for progress is also automated to ensure a systematic approach to rehabilitation. In NEC, the task difficulty increases monotonically with consecutive training sessions. This is achieved by changing the set-size and target/distractor similarity. Other devices such as Bioness Integrated Therapy Systems (BIONES Inc., USA) have also attempted to modify task difficulty by using word searches. However, as reading difficulties are often comorbid with visual field defects [19, 21], this approach confounds hemianopia and dyslexia, making it less appropriate for a large proportion of patients.

In clinical practice, improving a patient's condition or alleviating the disability is of prime importance. In the absence of a standardised approach, clinical tools stated above are used by occupational therapists and rehabilitation workers to help the patients and improve their interactions with their immediate environment. NeuroEyeCoach is specifically designed for compensatory therapy, it is evidence based and relies on a body of previous studies showing improved eye movement efficiency in hemianopic patients. We have demonstrated that it is on a par with its earlier version used previously in clinical settings. Importantly, as it is web-deliverable, it can also be used unsupervised in the home environment.

## Figures and Tables

**Table 1:** Studies using visual search task as an intervention to improve eye movement in hemianopia.

Study	Number of patients	Session duration (minutes)	Total training duration (hours)
Zihl, 1988 [11]	30	15–45	11.25
Kerkhoff et al., 1992 [13]	92	20–30	10
Zihl, 1995 [6]	14	14–45	10.5
Nelles et al., 2001 [14]	21	30–40	20
Pambakian et al., 2004 [15]	29	20–40	13.3
Nelles et al., 2009 [16]	11	20–30	10
Roth et al., 2009 [17]	14	30	30
Mannan et al., 2010 [18]	29	40	26.7
Zihl, 2011 [19]	157	10–45	7.5
Mödden et al., 2012 [20]	45	15–30	7.5
Schuett et al., 2012 [21]	36	12–45	9
Schuett and Zihl, 2013 [22]	38	11–45	8.25
Lane et al., 2010 [23]	42	40	10
Aimola et al. 2014 [24]	52	35–60	35

**Table 2:** Outcome of assessments with the cancellation and the visual search tasks in the experimental (EG) and in the control groups (CG) (means in seconds; SD in brackets). For comparison, normal subjects required on average 13.2 s (SD: 1.3; *n* = 25) for completing the cancellation task and 0.64 s (SD = 0.2; *n* = 10) for the visual search task.

Task	Assessment 1	Assessment 2	Assessment 3	Assessment 4
Cancellation				
EG	38.1 (12.9)	35.1 (11.9)	24.4 (8.3)	25.1 (7.1)
CG	40.0 (11.4)	39.8 (10.7)	39.4 (11.3)	
Visual search				
EG	1.42 (0.7)	1.39 (0.7)	1.02 (0.5)	0.98 (0.5)
CG	1.47 (0.8)	1.46 (0.6)	1.45 (0.6)	

**Table 3:** Subjective reports for experimental (EG) group at assessments 2, 3, & 4 and the control group at assessments 2 & 3.

Category	Assessment 2	Assessment 3	Assessment 4
Vision too slow			
EG	16 (100%)	4 (40%)	2 (12.5%)
CG	16 (100%)	14 (81.3%)	
Bumping into obstacles			
EG	6 (37.5%)	1 (6.3%)	0
CG	7 (43.8%)	5 (31.3%)	
Getting lost			
EG	3 (18.8%)	1 (6.3%)	0
CG	2 (12.5%)	1 (6%)	
